# Soil pH enhancement and alterations in nutrient and Bacterial Community profiles following *Pleioblastus amarus* expansion in tea plantations

**DOI:** 10.1186/s12870-024-05374-0

**Published:** 2024-09-06

**Authors:** Lili Fan, Shuanglin Chen, Ziwu Guo, Ruicai Hu, Liangjin Yao

**Affiliations:** 1 Research Institute of Subtropical Forestry, Chinese Academy of Forestry, Hangzhou, 311400 China; 2 Zhejiang Academy of Forestry, Hangzhou, 310023 China; 3 Longyou County Forestry Technology Extension Station, Quzhou, 324400 China

**Keywords:** Bamboo forests expansion, Tea plantations, Soil pH, Soil nutrients, Soil bacteria

## Abstract

**Background:**

The expansion of bamboo forests increases environmental heterogeneity in tea plantation ecosystems, affecting soil properties and microbial communities. Understanding these impacts is essential for developing sustainable bamboo management and maintaining ecological balance in tea plantations.

**Methods:**

We studied the effect of the continuous expansion of *Pleioblastus amarus* into tea plantations, by establishing five plot types: pure *P. amarus* forest area (BF), *P. amarus* forest interface area (BA), mixed forest interface area (MA), mixed forest center area (TB), and pure tea plantation area (TF). We conducted a comprehensive analysis of soil chemical properties and utilized Illumina sequencing to profile microbial community composition and diversity, emphasizing their responses to bamboo expansion.

**Results:**

(1) Bamboo expansion significantly raised soil pH and enhanced levels of organic matter, nitrogen, and phosphorus, particularly noticeable in BA and MA sites. In the TB sites, improvements in soil nutrients were statistically indistinguishable from those in pure tea plantation areas. (2) Continuous bamboo expansion led to significant changes in soil bacterial diversity, especially noticeable between BA and TF sites, while fungal diversity was unaffected. (3) Bamboo expansion substantially altered the composition of less abundant bacterial and fungal communities, which proved more sensitive to changes in soil chemical properties.

**Conclusion:**

The expansion of bamboo forests causes significant alterations in soil pH and nutrient characteristics, impacting the diversity and composition of soil bacteria in tea plantations. However, as expansion progresses, its long-term beneficial impact on soil quality in tea plantations appears limited.

**Supplementary Information:**

The online version contains supplementary material available at 10.1186/s12870-024-05374-0.

## Introduction

Bamboo expansion is a globally widespread natural phenomenon, notably conspicuous in regions of Asia, the Americas, and Africa [1–4]. Owing to its rapid growth and reproductive capacity, bamboo can swiftly colonize large areas, exerting a significant influence on local ecological balance [3, 5–7]. In natural ecosystems, bamboo expansion can compress indigenous plant communities and facilitate extensive bamboo forests, thus modifying the structure and functionality of ecosystems [8]. The ecological risks linked to bamboo expansion encompass diminished species diversity and the deterioration of indigenous forest ecosystems, potentially leading to decreased biodiversity and the incidence of severe diseases or insect infestations [1, 9, 10]. Alterations in plant characteristics during bamboo expansion, encompassing plant morphology, stand density, net primary production, litter quality, and root exudates, are recognized as principal factors shaping soil ecosystems [2, 11, 12]. Substantial changes in factors such as light, nutrients, water, spatial structure, and competition at the interface of bamboo expansion, leading to heightened environmental heterogeneity and profound impacts on soil properties [13–15]. Consequently, a comprehensive comprehension of the ramifications of bamboo expansion on soil and its associated ecosystems, along with the subsequent development of effective management strategies, constitutes pivotal measures in safeguarding ecological equilibrium and fostering sustainable development.

Bamboo expansion has a wide-ranging impact on soil chemical properties, including pH, organic matter, and nutrient cycles [2, 16–19]. Typically, soil pH is higher in mixed forests than in pure bamboo forests [1], especially when expanding into acidic coniferous forests, which can elevate soil pH compared to broad-leaved forests [18]. Nevertheless, research findings on soil pH are contentious [20]. Alterations in soil pH can influence nutrient cycling, thereby modifying the structure and function of ecosystems [2, 21]. Bamboo expansion often results in increased soil organic matter content, particularly organic carbon [22, 23]. Studies indicate that bamboo expansion into broad-leaved forests significantly increases soil organic carbon content [24, 25], whereas expansion into coniferous forests places soil organic carbon levels between that of coniferous forests and bamboo forests [23]. Furthermore, bamboo expansion may impact the nitrogen and phosphorus cycles through mechanisms such as root exudates, litter decomposition, and plant residue decomposition [22, 23, 26–29]. For example, total nitrogen content in mixed forest soil decreases when bamboo expands into broad-leaved forests [19, 27], but shows no significant change when bamboo expands into pine-broadleaf mixed forests [29]. The rise in soil pH following bamboo expansion enhances phosphorus availability, resulting in significant differences between expanding coniferous and broad-leaved forests [1, 2, 29, 30]. These variations likely relate to the type of vegetation and the stage of bamboo expansion, but current research predominantly focuses on coniferous and broad-leaved forests, with limited documentation of bamboo expanding into other forest types.

Soil microorganisms are pivotal for nutrient cycling and soil fertility, exhibiting significant variability survival strategies and adaptation to environmental conditions [15]. Bamboo expansion modifies soil properties, which in turn influence microbial communities and potentially lead to changes in microbial abundance, composition, and diversity [31]. Among the various factors shaping microbial communities, soil pH emerges as a key determinant, influencing the abundance and structure of both fungi and bacteria [1, 31, 32]. Studies have demonstrated that soil pH profoundly impacts soil fungi, with their abundance declining as pH increases, whereas bacteria play a primary role in nitrogen mineralization in bamboo forest soil as pH rises [9]. Moreover, pH elevation facilitates changes in phosphorus-associated bacteria, enhancing phosphorus release [33, 34]. Various forest types exert distinct impacts on microbial communities. For instance, bamboo expansion into broad-leaved forests is associated with more pronounced fungal alterations [9, 15, 28], whereas expansion into coniferous forests leads to more prominent bacterial variations [22, 35, 36]. Thus, there is a need for further investigation to explore the impacts of bamboo expansion on fungal and bacterial populations, as well as their correlation with soil properties across diverse forest types.

In China, particularly in southern regions such as Zhejiang and Fujian provinces, tea is a critical economic crop, with the country leading the world in tea plantation area and production yield [37–39]. The spatial distribution and ecological environments of tea gardens and bamboo forests often overlap, facilitating the natural expansion of bamboo into tea plantations [37, 40]. In recent years, shifts in tea plantation management practices from intensive to extensive practices, driven by rising labor costs and insufficient management [37, 40], have fostered the expansion of bamboo into declining tea plantations. Prolonged monoculture of tea trees has led to numerous soil and environmental issues, including changes in soil structure and nutrients, environmental pollution, soil acidification, reduced microbial diversity, and severe soil erosion, which collectively threaten the economic viability and ecological stability of tea plantations [39, 41–43]. The expansion of bamboo introduces additional ecological factors, such as altered light conditions, soil properties, and spatial competition, further impacting the ecological balance of tea plantations. Despite existing research on bamboo coexistence primarily highlights the economic and ecological benefits of integrating tea trees under bamboo forests [43], the specific effects of bamboo expansion on tea plantation soil properties and microbial community characteristics require further clarification.

The expansion of *Pleioblastus amarus* (Keng) P. C. Keng in tea plantations poses significant challenges to soil health and microbial communities, necessitating thorough investigation. This study aims to elucidate the impact of *P. amarus* expansion on soil pH, nutrient levels, and the diversity and structure of microbial communities at the continuous expansion interface within tea plantations. By examining these factors, we seek to provide a scientific foundation for developing phased *P. amarus* control strategies and targeted measures to restore the ecological balance of tea plantations. Furthermore, this research contributes to a deeper understanding of bamboo expansion dynamics. We hypothesized that the expansion of *P. amarus* into tea plantations would lead to (1) significant changes in soil pH and nutrient levels, influenced by the stage of bamboo expansion; (2) notable alterations in soil microbial diversity and species composition, encompassing fungi and bacteria, also influenced by the stage of bamboo expansion; and (3) close associations between the composition and abundance of soil microbial communities and changes in soil nutrient patterns.

## Materials and methods

### Overview of the study site

The study site, located in Muchen Township, Longyou County, Zhejiang Province, China (119°13′25.88″E, 28°49′4.85″N), experiences a subtropical monsoon climate with distinct seasons. With an average annual rainfall of 1,620 mm and a mean temperature of 17.40 °C, the region boasts a frost-free period lasting 261 days on average. The relative humidity hovers around 79%, while the annual sunshine duration extend to 1,769 h. Characterized by red loam soil ranging from 70 to 100 cm in depth, the soil exhibits a pH of 4.56 and an organic matter content of 37.18 g·kg^− 1^. Notably, soil nitrogen, phosphorus, and potassium content measure 1.81 g·kg^− 1^, 0.51 g·kg^− 1^, and 22.52 g·kg^− 1^, respectively. Established in 1,972, the tea plantation initially focused on cultivating varieties such as Longjing green tea. However, since 2008, the plantation has undergone a gradual decline, shifting from intensive to extensive management practices. Consequently, naturally occurring *Pleioblastus amarus* (Keng) P. C. Keng forests (Clonal breeding), untouched by human intervention, have encroached upon the original tea plantation area. Currently, *P. amarus* forests spans approximately 1 hm^2^, while the tea plantation covers 0.53 hm^2^.

### Experimental design

**Fig. 1:**
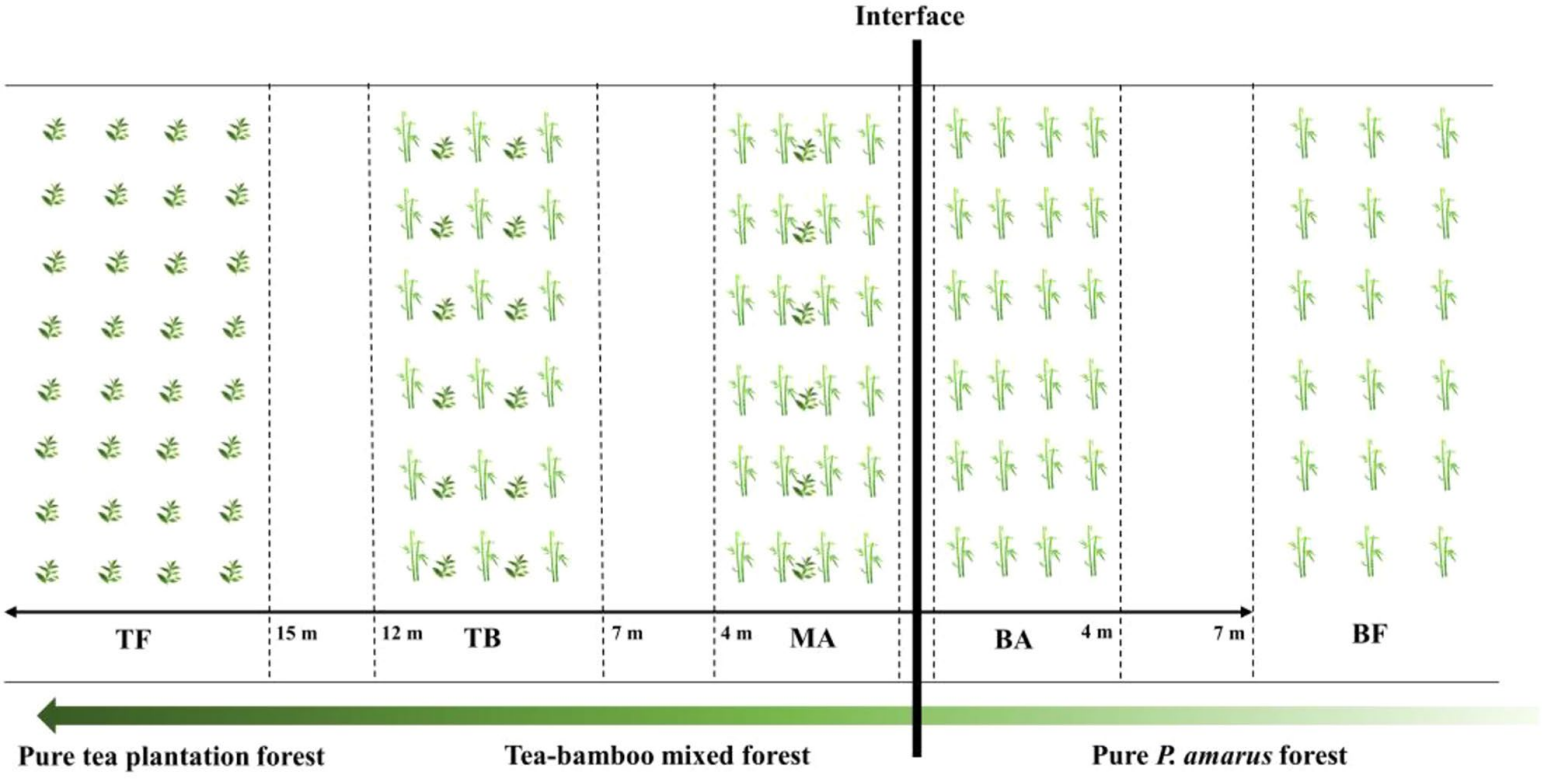
Schematic illustration of sample sites depicting *Pleioblastus amarus* expansion into tea plantations

We established sampling sites along the boundary line between bamboo and tea trees, encompassing two types of forest stands: pure *P. amarus* forest and mixed forest, characterized by similar site conditions. Five types of sampling sites were designated: pure *P. amarus* forest area (BF), *P. amarus* forest interface area (BA), mixed forest interface area (MA), mixed forest center area (TB), and pure tea plantation area (TF) (Fig. 1). Each sample plot had a strip length of approximately 20 m within the 12 m width of the expanding *P. amarus* area. The spacing between each sample plot exceeded 3 m, except for the interface area, to ensure spatial independence. At each site, we established three sampling quadrats measuring 3 m × 3 m.

We conducted comprehensive field investigations, including measurements of the height and diameter at breast height of all standing *P. amarus* and the height and crown width of tea trees in each site. Additionally, we calculated *P. amarus* density and stand density and recorded topographic conditions such as slope, aspect, and altitude (Supplementary Table S1). Human intervention varied across sites, with BF, BA, MA, and TB sites left under conditions of no human intervention, while in TF site tea trees underwent minor pruning, reflecting common management practices in tea plantations.

Soil samples were collected from 0 to 30 cm depth using the diagonal method in each quadrat. Approximately 1 kg of soil sample was obtained using the quartering method, with part of the sample immediately stored at -80 °C in the laboratory after removing roots, rocks, weeds, and other debris, for subsequent high-throughput sequencing of soil fungi and bacteria. Another part of the soil sample was air-dried naturally, sieved through a 2 mm, and processed for pH and available nutrient determination, with additional sieving through a 0.15 mm for total nutrient determination.

### Measurement indices and methods

#### Determination of soil chemical properties

The soil pH was measured using a pH meter (PHS-3E, Shanghai Yidian Scientific Instrument Co., Ltd., China) at a soil-to-water ratio of 1:2.5. Soil total nitrogen (TN) content was analyzed using an elemental analyzer (Elementar Vario, C/N analyzer, Germany) [44]. Soil total organic carbon was determined via the concentrated sulfuric acid-potassium dichromate heating method, and soil organic matter (OM) was calculated by multiplying the total soil carbon by a conversion factor of 1.72 [45]. Soil total phosphorus (TP) content was assessed using an alkali fusion method, and soil total potassium (TK) content was measured via an acid dissolution method. The soil hydrolyzable nitrogen (HN) content was evaluated using an alkali diffusion method. Available phosphorus (AP) content was determined using the NaHCO_3_ extraction method coupled with the molybdenum antimony anti-colorimetric method, and available potassium (AK) content was determined using flame photometry [46]. Each parameter was evaluated with triplicate biological replicates.

#### High-throughput sequencing of soil microbial communities

Soil genomic DNA extraction was conducted using the E.Z.N.A. Soil DNA Kit (Omega Bio-tek, Inc., USA) according to the manufacturer’s instructions. The concentration and quality of the genomic DNA were assessed using a NanoDrop 2000 spectrophotometer (Thermo Scientific Inc., USA). Subsequently, DNA samples were stored at -20°C for further experimentation. The V3-4 hypervariable region of the bacterial 16S rRNA gene was amplified using the universal primers 338F (5’-ACTCCTACGGGAGGCAGCAG-3’) and 806R (5’-GGACTACNNGGGTATCTAAT-3’). Similarly, the internal transcribed spacer 1 (ITS1) region of the fungal ribosomal RNA (rRNA) gene was targeted using the primers ITS1 (5’-CTTGGTCATTTAGAGGAAGTAA-3’) and ITS2 (5’-TGCGTTCTTCATCGATGC-3’). Deep sequencing was performed on the Illumina Miseq/Novaseq platform (Illumina, Inc., USA) at Beijing Allwegene Technology Co., Ltd. Following the sequencing run, image analysis, base calling, and error estimation were carried out using the Illumina Analysis Pipeline Version 2.6 (Illumina, Inc., USA). Sequence data associated with this project have been deposited in the NCBI Short Read Archive database (Accession Number: CRA016300).

### Data processing

One-way ANOVA was utilized to assess differences in soil properties and α-diversity among various sites, with post-hoc multiple comparisons conducted using the Duncan method (*p* < 0.05). Statistical data were expressed as mean ± standard error (SE). All ANOVA analysis were performed using SPSS 22.0 software (IBM Corporation, Armonk, NY, USA). Alpha (α) diversity analysis was conducted using QIIME (v1.8.0) software, calculating the richness index (Chao1), the number of observed Operational Taxonomic Units (OTUs) (observed species), phylogenetic diversity (PD_whole_tree), and Shannon index [47]. Venn diagrams were generated using the “Vegan” package in R language (v4.2.1). Beta (β) diversity distance matrices were calculated using QIIME (v2.0.0), and principal component analysis (PCA), principal co-ordinates analysis (PCoA), and partial least squares discriminant analysis (PLS-DA) were performed using R software based on Euclidean distance. LEfSe (LDA Effect Size) analysis was employed to examine differences in species abundance among treatments using Python (v2.7), with an LDA threshold set at 4.0 [48]. Dominant genera with a relative abundance greater than 0.1% were identified and compared between groups for significant differences. Redundancy analysis (RDA) and Pearson correlation analysis were conducted to explore the relationship between key microorganisms (OUTs) at the phylum level and soil chemical properties. RDA and chord diagrams were created using the “vegan” and “Circle” package in R, respectively [39].

## Result

### Changes in soil chemical properties across various sampling sites following the expansion of *P. Amarus* in tea plantations

After the expansion of *P. amarus* into tea plantations, distinct trends in soil chemical properties emerged (Table 1). Soil pH, OM, TN, TP, HN, AP, and AK displayed an upward trend, with the highest levels recorded in the MA site and the lowest in the TF site. Conversely, TK content notably declined along the expansion gradient from *P. amarus* to tea plantations (*p* < 0.05). In BA, MA, and TB sites, soil pH significantly exceeded that of BF and TF sites (*p* < 0.05). Additionally, OM contents significantly surpassed those of TF sites in BA and MA sites (*p* < 0.05), whereas soil TN and TP contents in BA and MA sites were notably higher than those in TB and TF sites (*p* < 0.05). No significant disparities in these four soil properties were observed between BA and MA sites and BF site. Furthermore, TK content markedly exceeded those of TB and TF sites in BF, BA, and MA sites (*p* < 0.05). AK content in the MA site significantly surpassed that of other sites (*p* < 0.05). Remarkably, the expansion of *P. amarus* had no significant impact on soil HN and AP content in the tea plantations.

**Table 1:** Soil chemical properties across various sampling sites after the expansion of *Pleioblastus amarus* within tea plantations

Sampling Sites	pH	OM /(g·kg^− 1^)	TN /(g·kg^− 1^)	TP /(g·kg^− 1^)	TK /(g·kg^− 1^)	HN /(mg·kg^− 1^)	AP /(mg·kg^− 1^)	AK /(mg·kg^− 1^)
BF	4.52 ± 0.01b	37.97 ± 0.81ab	1.85 ± 0.05ab	0.54 ± 0.02a	26.03 ± 0.87a	145.80 ± 6.11a	1.73 ± 0.21a	181.38 ± 2.18b
BA	4.61 ± 0.01a	39.83 ± 1.02a	1.93 ± 0.05a	0.55 ± 0.01a	25.28 ± 0.74a	146.43 ± 10.75a	2.48 ± 0.32a	192.63 ± 0.13ab
MA	4.66 ± 0.01a	40.30 ± 0.74a	2.01 ± 0.03a	0.55 ± 0.01a	23.95 ± 1.19a	154.46 ± 10.71a	3.20 ± 0.53a	201.72 ± 5.93a
TB	4.61 ± 0.01a	35.60 ± 1.19ab	1.70 ± 0.07bc	0.47 ± 0.07b	18.33 ± 0.36b	157.95 ± 6.54a	2.67 ± 0.31a	185.94 ± 0.17b
TF	4.37 ± 0.04c	32.17 ± 2.05b	1.57 ± 0.08c	0.46 ± 0.02b	18.97 ± 0.38b	123.38 ± 6.41a	1.88 ± 0.09a	161.00 ± 2.28c

*Note*, Different lowercase letters in the same column indicate significant differences at the 0.05 level. The sampling sites are categorized as follows: BF (pure P. amarus forest center area) BA (P. amarus forest interface area) MA (mixed forest center area) TB (mixed forest interface area) and TF (pure tea plantation area)

### Soil Microbial diversity and composition across various sampling sites following the expansion *P. Amarus* in tea plantations

**Fig. 2:**
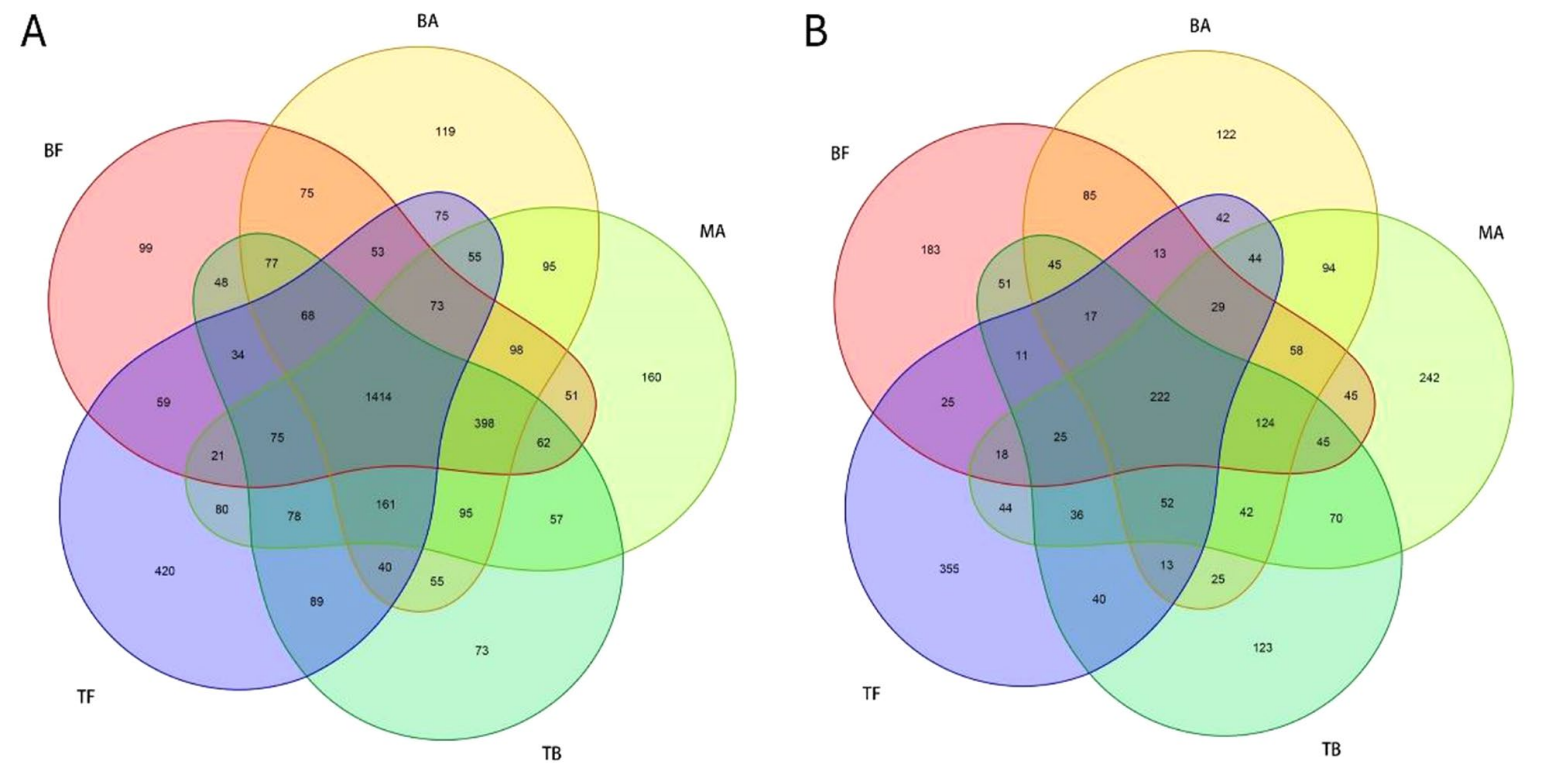
Venn diagram of soil OTUs across various sampling sites after the expansion of *P. amarus* within tea plantations. *Note* Panels A and B depict the unique and overlapping OTUs of bacterial and fungal communities, respectively, in soil samples from various sampling sites. The sampling sites are categorized as follows: BF (pure *P. amarus* forest center area), BA (*P. amarus* forest interface area), MA (mixed forest center area), TB (mixed forest interface area), and TF (pure tea plantation area)

Sequencing of ITS and 16 S rRNA genes produced 1,261,933 and 1,421,916 clean reads, respectively. The associated bacteria were classified into 2 kingdoms, 34 phyla, 83 classes, 190 orders, 286 families, 488 genera, and 406 species, whereas the associated fungi were categorized into 1 kingdom, 13 phyla, 45 classes, 119 orders, 233 families, 447 genera, and 679 species. The soil microbial communities across the five sampling sites exhibited 1,414 shared bacterial OTUs, with 119 (BA site), 99 (BF site), 160 (MA site), 73 (TB site), and 420 (TF site) specific bacterial OTUs (Fig. 2A), and 222 shared fungal OTUs, with 122 (BA site), 183 (BF site), 242 (MA site), 123 (TB site), and 355 (TF site) specific fungal OTUs (Fig. 2B).

**Fig. 3:**
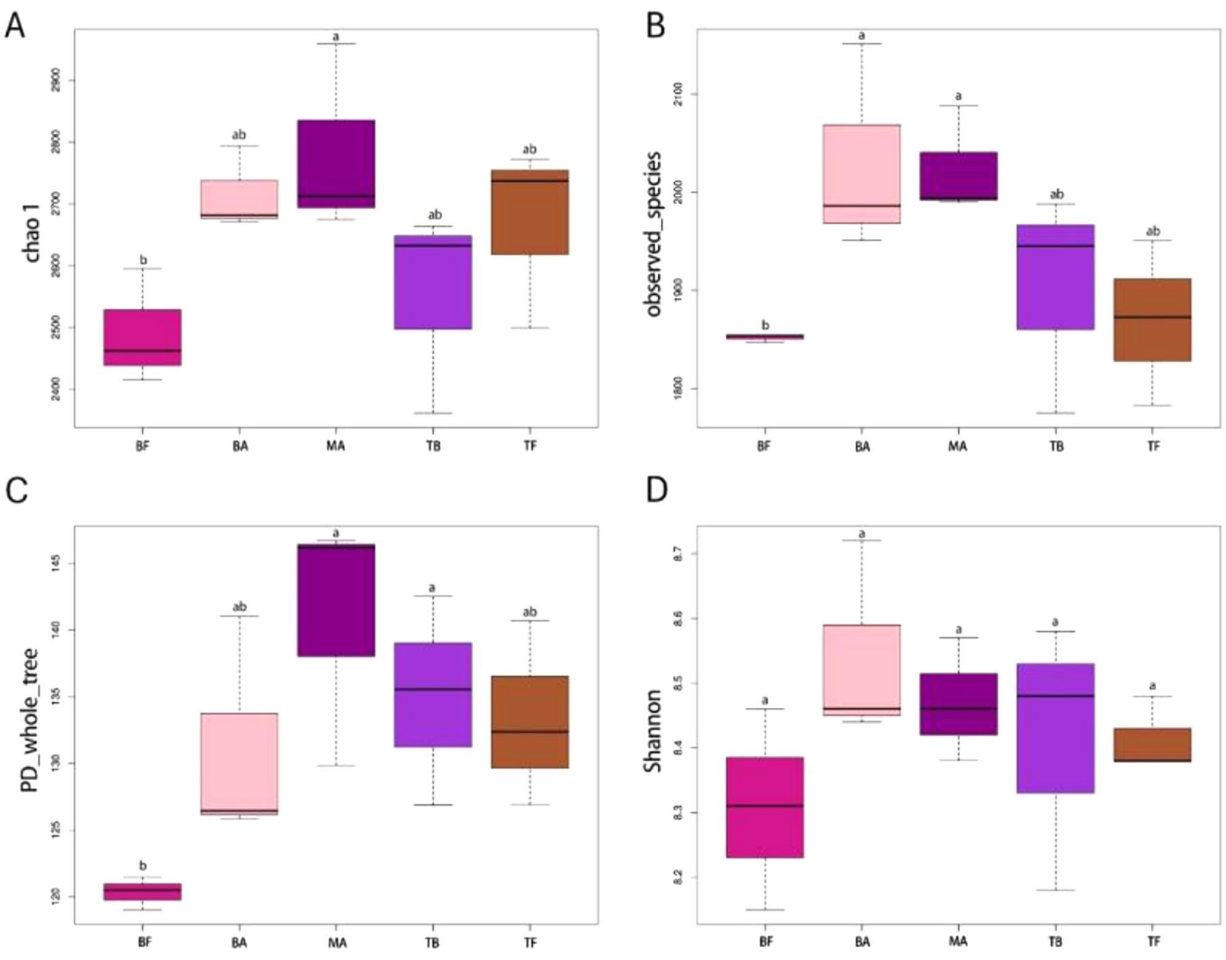
Soil bacterial α-diversity across various sampling sites after the expansion of *P. amarus* within tea plantations. *Note* Panels A, B, C, and D represent bacterial Chao1 index, observed_species, PD_whole_tree, and Shannon index, respectively. Different lowercase letters indicate significant differences in various sampling sites at the 0.05 level. The sampling sites are categorized as follows: BF (pure *P. amarus* forest center area), BA (*P. amarus* forest interface area), MA (mixed forest center area), TB (mixed forest interface area), and TF (pure tea plantation area)

**Fig. 4:**
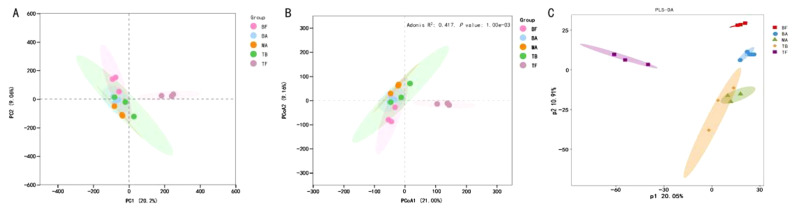
Soil bacterial β-diversity across various sampling sites after the expansion of *P. amarus* within tea plantations. *Note* Panels A, B, and C depict the results of PCA, PCoA, and PLS-DA analyses of bacterial OTUs across various sampling sites, respectively. Different colored or shaped dots indicate different sample groups: BF (pure *P. amarus* forest center area), BA (*P. amarus* forest interface area), MA (mixed forest center area), TB (mixed forest interface area), and TF (pure tea plantation area)

Microbial community diversity is assessed by α-diversity (Fig. 3). Following *P. amarus* expansion, a significant increase in the Chao1 index of bacterial communities in tea plantation soils was observed (*p* < 0.05), whereas the observed_species and PD_whole_tree indices exhibited a pattern of increase followed by a decrease. The MA site had the highest Chao1 and PD_whole_tree indices among all sites, significantly differing from the BF site (*p* < 0.05). Similarly, the observed_species index peaked at BA and MA sites, significantly differing from the BF site (*p* < 0.05). Nonetheless, there were no significant differences in the Shannon diversity index of bacterial and fungal communities after the expansion of *P. amarus* (Supplementary Figure S1). To further analyze the differences between groups, β diversity was examined (Fig. 4 and Supplementary Figure S2). PCA and PCoA analyses of both bacterial and fungal communities showed distinct separation among the BF, BA, MA, TB, and TF groups, indicating significant differences in soil microbial communities following the expansion of *P. amarus* into tea plantations. Additionally, the PLS-DA model confirmed significant differences among the five groups.

**Fig. 5:**
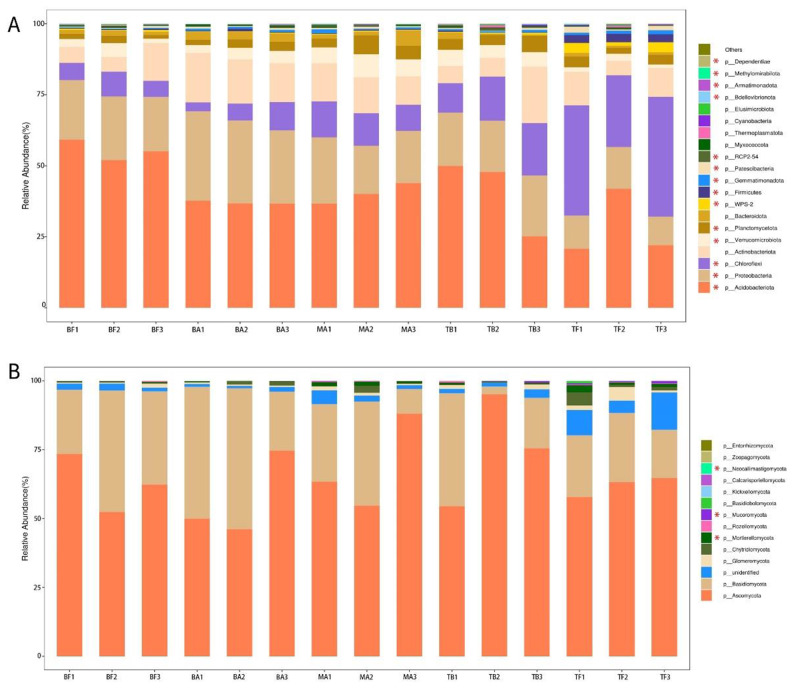
Composition of soil bacterial and fungal communities at the phylum level across various sampling sites after the expansion of *P. amarus* within tea plantations. *Note* Panel A illustrates the composition of bacterial communities at the phylum level, while Panel B depicts the composition of fungal communities at the phylum level. Asterisks (*) indicates significant differences determined by Duncan’s test (*p* < 0.05). The sampling sites are categorized as follows: BF (pure *P. amarus* forest center area), BA (*P. amarus* forest interface area), MA (mixed forest center area), TB (mixed forest interface area), and TF (pure tea plantation area)

Bacterial communities in BF, BA, MA, and TB sites were predominantly classified under the phylum *Acidobacteriota* (Fig. 5A). In contrast, *Chloroflexi* was the dominant phylum in the TF site, followed by *Acidobacteriota*. The expansion of *P. amarus* into tea plantations resulted in a notable decrease in the relative abundance of *Acidobacteriota* (*p* < 0.05) and a concurrent significant increase in the relative abundance of *Chloroflexi* (*p* < 0.05). *Ascomycota* (65.09%) and *Basidiomycota* (28.18%) constituted the predominant fungal phyla across the five sampling sites (Fig. 5B), collectively representing over 90% of the total fungal abundance. Although the composition of soil fungal communities remains consistent across all sites, significant differences were observed in the relative abundances of *Mortierellomycota*, *Mucoromycota*, and *Neocallimastigomycota* (*p*<0.05).

### The RDA and correlation analysis of key microorganisms and soil chemical properties across various sampling sites following the expansion of *P. Amarus* in tea plantations

**Fig. 6:**
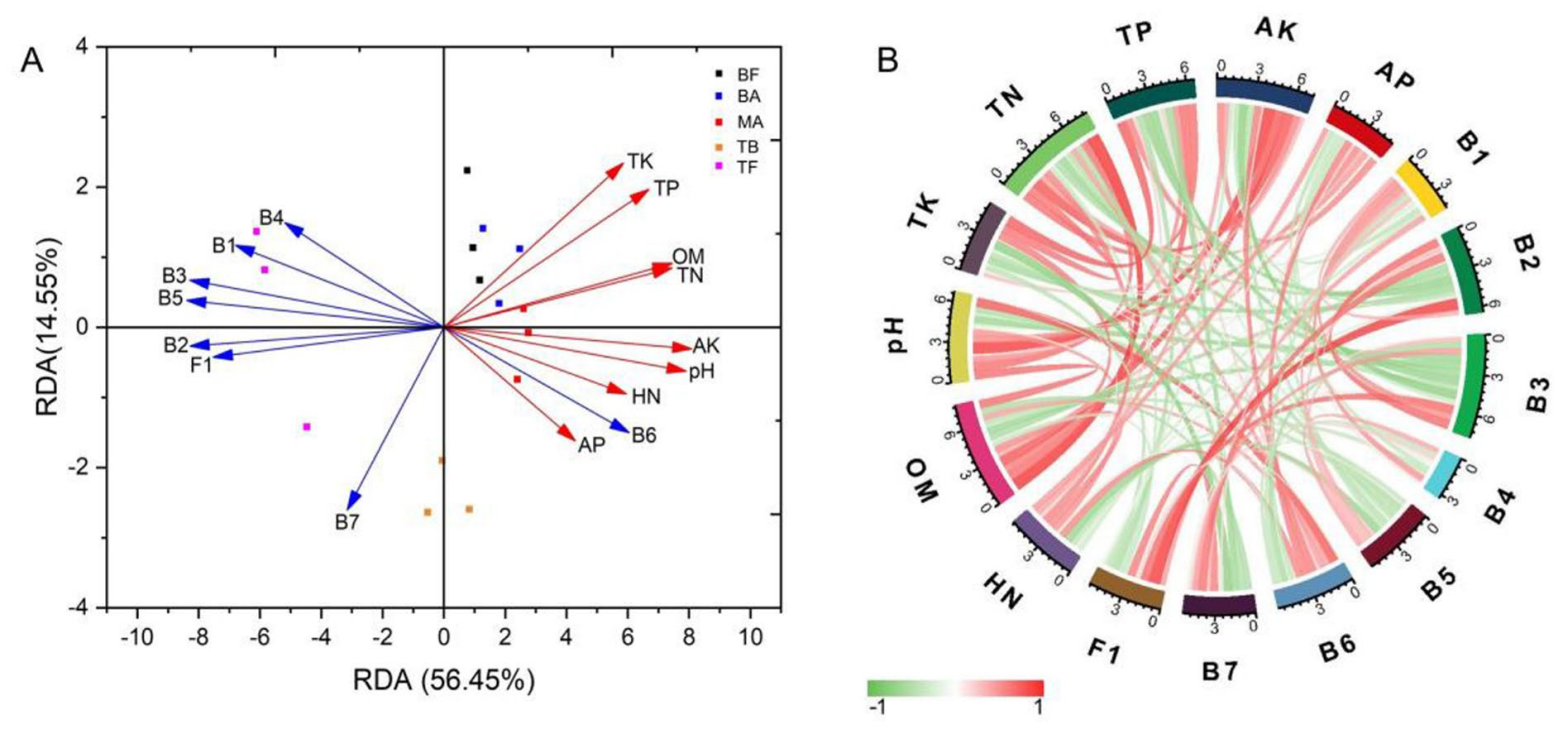
The relationship of soil chemical properties and key microorganisms (OUTs) across various sampling sites after the expansion of *P. amarus* within tea plantations. *Note* A and B represent PCA and chord diagram between soil chemical properties and the distribution of key microorganisms (OUTs) at the phylum level respectively. F1: *Mucoromycota*, B1: *Patescibacteria*, B2: *Chloroflexi*, B3: *WPS-2*, B4: *Bdellovibrionota*, B5: *Firmicutes*, B6, *Verrucomicrobiota*, B7: *Methylomirabilota*, OM: organic matter, HN: hydrolytic nitrogen, AP: available P, AK: available K, TK: total K, TP: total P. Red and green denote positive and negative differences between the sampling sites, respectively. Different colored dots indicate different sample groups: BF (pure *P. amarus* forest center area), BA (*P. amarus* forest interface area), MA (mixed forest center area), TB (mixed forest interface area), and TF (pure tea plantation area)

We conducted RDA and correlation analysis to examine the relationship between soil chemical properties and the distribution of OTUs at the phylum level. The findings revealed significant associations between soil chemical properties and microbial community at this taxonomic level. Specifically, statistically significant correlations were identified for one fungal phylum and seven bacterial phyla in relation to soil chemical properties (Fig. 6, Supplementary Table S2). The RDA results (Fig. 6A) demonstrated that the OTUs distribution in the BF, BA and MA sites was strongly influenced by soil chemical properties. Notably, *Verrucomicrobiota* has exhibited the strongest positive correlation with these properties. Conversely, the OTUs distribution in the TF sites showed a negative correlation with soil chemical properties. In the TB sites, there was no significant correlation observed between the microbial community and soil chemical properties. Detailed analysis using a correlation string graph revealed specific relationships at the phylum level (Fig. 6B). The fungus *Mucoromycota* exhibited a negative correlation with OM (*p* < 0.05). Among bacteria, *Patescibacteria* showed a negative correlation with HN (*p* < 0.05), while *Chloroflexi* displayed negative correlations with OM (*p* < 0.05). Additionally, *WPS-2* exhibited negative correlations with pH, AK, and OM (*p* < 0.05). *Bdellovibrionota* was negatively correlated with HN (*p* < 0.05). *Firmicutes* showed negative correlations with pH and HN (*p* < 0.05), whereas *Verrucomicrobiota* demonstrated positive correlations with pH and AP (*p* < 0.05). Lastly, *Methylomirabilota* displayed negative correlations with TP and TK (*p* < 0.05). No significant correlation was observed between TN and fungi and bacteria at the phylum level.

## Discussion

### The impact of *P. amarus* expansion on tea plantation soil chemical properties

The expansion of *P. amarus* has induced alterations in the soil chemical properties of tea plantations, signifying its impacts on the ecological environment. Soil pH in the BA, MA, and TB sites significantly exceeded that of BF and TF sites, implying a reduction in soil acidity following *P. amarus* expansion. The rise in soil pH could be linked to the potential stimulation of silicate mineral weathering rates within the bamboo forest triggered by its expansion [1, 49, 50], which consumes carbon dioxide and hydrogen ions, releases soluble silicon and alkaline ions, thus diminishing proton concentration, and thus elevating soil pH [51, 52]. This finding aligns with previous research on bamboo forests encroach into coniferous forests [16–19]. Nonetheless, in comparison to the expansion of Moso bamboo (*Phyllostachys edulis* (Carrière) J. Houz) forests [21, 24, 53], the impact of *P. amarus* expansion on soil pH in tea plantations appears relatively minor. This discrepancy warrants further investigation to understand species-specific effects on soil chemistry [1]. In addition to changes in pH, soil OM contents in BA and MA sites surpass those in BF and TF sites, indicating that *P. amarus* expansion has bolstered the humification process of organic matter, likely attributed to accelerated litter decomposition facilitated by highly active charcoal in bamboo forests [27, 54]. These results are consistent with the findings of Wang et al. [23] and Qin et al. [25], who reported increased soil organic carbon storage due to bamboo expansion.

The expansion of *P. amarus* into tea plantations has led to significant fluctuations in the soil TN and TP dynamics. At the interface zone (BA and MA sites), TN and TP levels were markedly higher compared to BF and TF sites. This disparity may arise from the heightened density of *P. amarus* in the interface zone, which results in more litter accumulation and rapid decomposition, consequently expediting the biological decomposition and release of soil N and P [55, 56]. In contrast, Song et al. [27] reported that the expansion of Moso bamboo into broad-leaved forests decelerates the soil N cycle. Similarly, Wu et al. [29] observed that found a significant decline in soil TP content following bamboo forest expansion. These discrepancies may be attributed to differences the vegetation types involved in the studies, indicating the need for further research. Moreover, this investigation revealed a decline in OM, OC, TN, and TP levels in the TB site relative to BA and MA sites. This reduction could be attributed to the decreased bamboo density at the forefront of expansion, despite increases in bamboo diameter at breast height and height (Supplementary Figure Table S1), which led to robust bamboo growth and enhanced soil nutrient absorption [28, 57]. Moreover, intensified competition between bamboo forests and tea trees likely contributed to a reduction in soil nutrient content. This observation underscores the disparities in the dynamic changes in soil chemical properties during the expansion of bamboo forests into tea plantations.

### The impact of *P. Amarus* expansion on tea plantation on soil microbial diversity and structure

Following the expansion of *P. amarus*, *Acidobacteria* emerged as the predominant bacterial phylum across the BF, BA, MA, and TB sites, aligning with findings from previous findings that *Acidobacteria* adapt well to acidic soils and low pH levels [1, 35, 36, 58–60]. In contrast, *Chloroflexi* were more prevalent in TF sites, likely attributed to their compatibility with the rhizosphere microenvironment of tea trees [61, 62], including resilience to low pH and efficient organic matter utilization [63]. Despite their adaptability to acidic conditions, the relative abundance of *Acidobacteria* significantly declined, indicating that other environmental or competitive factors significantly impact their distribution and growth. Research indicates that bamboo forest expansion increases soil pH, which supports the proliferation of specific fungi [1, 28, 63, 64]. As soil pH increases, the relative abundance of *Ascomycota* and *Basidiomycota* also increases [28, 63, 64] became the dominant fungal phyla in tea plantations after *P. amarus* expansion [15, 19]. However, *P. amarus* expansion didn’t markedly affect these fungi. Instead, notable shifts in the presence of low-abundance *Mortierellomycota*, *Mucoromycota*, and *Neocallimastigomycota* were observed, which are crucial for organic matter decomposition, nutrient cycling, and plant nutrient uptake [65–67]. Thus, bamboo forest growth may modify soil ecosystem functions and the ecological roles of these fungi, with lower-abundance fungi facing increased competition and undergoing significant shifts during *P. amarus*’s expansion.

### The Interrelationship Between Soil Microbial Community Composition and Soil Chemical Properties in Tea Plantations During the Expansion of* P. Amarus*

The RDA and correlation analysis graphs illustrate the relationships between soil chemical properties and microbial communities across different sampling sites during various stages of *P. amarus* expansion in tea plantations. Changes in soil chemical properties significantly impacted the microbial community structure in the BF, BA, and MA sites. These findings corroborate prior research indicating that pH acts as the primary determinant in the alterations of microbial community configurations during bamboo forest expansion [1, 18, 28, 35]. Soil pH exhibited a negative correlation with lower-abundance bacterial groups such as *WPS-2*, *Firmicutes*, and *Methylomirabilota*, while showing positive correlations with *Verrucomicrobiota*. This suggests that bamboo expansion may reduce the habitat suitability for acidophilic bacteria, especially adversity-resistant *Firmicutes* [68]. Although certain bacterial groups, including *Patescibacteria*, *Bdellovibrionota*, and *Firmicutes*, may have competitive advantages or reduced nitrogen requirements in low-nitrogen settings, the impact of bamboo expansion on soil nitrogen cycling appears minimal due to the negligible variation in soil HN content.

Moreover, the increase in soil pH could boost phosphate-solubilizing bacteria activity, facilitating available phosphorus mobilization [30, 33, 34]. The study indicates that *Verrucomicrobiota* positively influences pH and AP during bamboo expansion, though its role in the broader phosphorus cycle remains marginal. In fungal communities, the response to soil pH was less pronounced. However, their dynamics were closely tied to the quantity and quality of available soil carbon and organic matter [15, 69, 70]. Lower-abundance *Mucoromycota* fungi and bacterial groups such as *Patescibacteria*, *Chloroflexi*, and *WPS-2* exhibited negative correlations with elevated soil OM levels, suggesting these microorganisms might play a more significant role in organic matter decomposition under conditions of low organic matter (e.g. BF and TF sites), highlighting their competitive disadvantage. Conversely, dominant soil microorganisms thrive in OM-rich environments after the expansion of *P. amarus* within tea plantations, intensifying the competitive strain on less abundant counterparts.

## Conclusions

The expansion of *P. amarus* within tea plantations markedly elevates soil pH, organic matter, organic carbon, and the contents of nitrogen and phosphorus. These changes lead to notable shifts in soil bacterial diversity and alterations in the composition of bacterial and fungal communities, with less abundant microbial taxa showing heightened sensitivity to the changing soil chemical properties. Given these impacts, strategies such as controlled planting and nutrient management are recommended to prevent further expansion of *P. amarus*. Alternatively, the enhanced soil nutrients can be utilized to improve tea tree growth. After a long expansion, efforts should focus on ecological restoration practices, including reforestation with native species and soil amendment techniques, to ensure the long-term sustainability of tea plantations. Future research should explore the long-term ecological consequences of bamboo expansion and develop precise management practices to mitigate adverse effects while leveraging potential benefits. Integrating these findings with broader literature will provide a comprehensive understanding of bamboo-ecosystem interactions and inform sustainable agricultural practices.

## Electronic supplementary material

Below is the link to the electronic supplementary material.

Supplementary Material 1

## Data Availability

The datasets generated and/or analyzed during the current study are available in the NGDC repository, accessible via CRA016300 (https://ngdc.cncb.ac.cn/gsa/s/EvvsLNHb).

## References

[1] Wu Y, Guo J, Tang Z, Wang T, Li W, Wang X, et al. Moso bamboo (Phyllostachys edulis) expansion enhances soil pH and alters soil nutrients and microbial communities. Sci Total Environ. 2024;912:169346. 38097081 10.1016/j.scitotenv.2023.169346

[2] Xu QF, Liang CF, Chen JH, Li YC, Qin H, Fuhrmann JJ. Rapid bamboo invasion (expansion) and its effects on biodiversity and soil processes. Glob Ecol Conserv. 2020;21:e00787.

[3] Bystriakova N, Kapos V, Lysenko I, Stapleton CMA. Distribution and conservation status of forest bamboo biodiversity in the Asia-Pacific Region. Biodivers Conserv. 2003;12:1833–41. 10.1023/A:1024139813651

[4] Griscom BW, Ashton PMS. A self-perpetuating bamboo disturbance cycle in a neotropical forest. J Trop Ecol. 2006;22:587–97. 10.1017/S0266467406003361

[5] Bai S, Wang Y, Conant RT, Zhou G, Xu Y, Wang N, et al. Can native clonal Moso bamboo encroach on adjacent natural forest without human intervention? Sci Rep. 2016;6:31504. 27600881 10.1038/srep31504 PMC5013281

[6] Bai S, Conant RT, Zhou G, Wang Y, Wang N, Li Y, et al. Effects of Moso bamboo encroachment into native, broad-leaved forests on soil carbon and nitrogen pools. Sci Rep. 2016;6:31480. 27526781 10.1038/srep31480 PMC4985758

[7] Suzuki S. Chronological location analyses of giant bamboo (Phyllostachys pubescens) groves and their invasive expansion in a satoyama landscape area, western Japan. Plant Species Biol. 2015;30:63–71. 10.1111/1442-1984.12067

[8] Liu W, Liao L, Liu Y, Wang Q, Murray PJ, Jiang X, et al. Effects of Phyllostachys pubescens expansion on underground soil fauna community and soil food web in a Cryptomeria japonica plantation, Lushan Mountain, subtropical China. J Soils Sediments. 2021;21:2212–27. 10.1007/s11368-021-02923-0

[9] Xu QF, Jiang PK, Wu J, Sen, Zhou GM, Shen RF, Fuhrmann JJ. Bamboo invasion of native broadleaf forest modified soil microbial communities and diversity. Biol Invasions. 2015;17:433–44. 10.1007/s10530-014-0741-y

[10] Griscom BW, Ashton PMS. Bamboo control of forest succession: Guadua sarcocarpa in Southeastern Peru. Ecol Manage. 2003;175:445–54. 10.1016/S0378-1127(02)00214-1

[11] Kubartová A, Moukoumi J, Béguiristain T, Ranger J, Berthelin J. Microbial diversity during cellulose decomposition in different forest stands: I. Microbial communities and environmental conditions. Microb Ecol. 2007;54:393–405. 17609845 10.1007/s00248-007-9286-2

[12] Ehrenfeld JG. Ecosystem consequences of Biological invasions. Annu Rev Ecol Evol Syst. 2010;41:59–80. 10.1146/annurev-ecolsys-102209-144650

[13] Guo Z, Lin H, Chen S, Yang Q. Altitudinal patterns of leaf traits and leaf allometry in bamboo Pleioblastus Amarus. Front Plant Sci. 2018;9. 10.3389/fpls.2018.01110 PMC6080356 30108603

[14] Xie Y, Zhang W, Guo Z, Du X, Fan L, Chen S, et al. Effects of vegetation succession on soil microbial stoichiometry in Phyllstachys edulis stands following abandonment. Sci Total Environ. 2023;895:164971. 37336394 10.1016/j.scitotenv.2023.164971

[15] Liu C, Zhou Y, Qin H, Liang C, Shao S, Fuhrmann JJ, et al. Moso bamboo invasion has contrasting effects on soil bacterial and fungal abundances, co-occurrence networks and their associations with enzyme activities in three broadleaved forests across subtropical China. Ecol Manage. 2021;498:119549. 10.1016/j.foreco.2021.119549

[16] Li C, Liu Y, Wang H, Chen Q, Deng B, Liu X, et al. Effects of moso bamboo (Phyllostachys edulis) expansion and simulated nitrogen deposition on emission of soil N2O and CO2 in Lushan Mountain. Acta Pedol Sin. 2019;56:146–55.

[17] Zhang M, Wang W, Bai SH, Xu Z, Yun Z, Zhang W. Linking Phyllostachys edulis (moso bamboo) growth with soil nitrogen cycling and microbial community of plant-soil system: effects of plant age and niche differentiation. Ind Crops Prod. 2022;177:114520. 10.1016/j.indcrop.2022.114520

[18] Umemura M, Takenaka C. Changes in chemical characteristics of surface soils in hinoki cypress (Chamaecyparis obtusa) forests induced by the invasion of exotic Moso bamboo (Phyllostachys pubescens) in central Japan. Plant Species Biol. 2015;30:72–9. 10.1111/1442-1984.12038

[19] Li YC, Li YF, Chang SX, Xu QF, Guo ZY, Gao Q, et al. Bamboo invasion of broadleaf forests altered soil fungal community closely linked to changes in soil organic C chemical composition and mineral N production. Plant Soil. 2017;418:507–21. 10.1007/s11104-017-3313-y

[20] Song QN, Yang QP, Liu J, Yu DK, Fang K, Xu P, et al. Effects of Phyllostachys edulis expansion on soil nitrogen mineralization and its availability in evergreen broadleaf forest. Chin J Appl Ecol. 2013;24:338–334. 23705376

[21] Zhang Y, Wang R, Sardans J, Wang B, Gu B, Li Y, et al. Resprouting ability differs among plant functional groups along a soil acidification gradient in a meadow: a rhizosphere perspective. J Ecol. 2023;111:631–44. 10.1111/1365-2745.14051

[22] Chang EH, Chiu CY. Changes in soil microbial community structure and activity in a cedar plantation invaded by moso bamboo. Appl Soil Ecol. 2015;91:1–7. 10.1016/j.apsoil.2015.02.001

[23] Wang HC, long Tian G, Chiu CY. Invasion of moso bamboo into a Japanese cedar plantation affects the chemical composition and humification of soil organic matter. Sci Rep. 2016;6:32211. 27558833 10.1038/srep32211 PMC4997307

[24] Yang C, Ni H, Zhong Z, Zhang X, Bian F. Changes in soil carbon pools and components induced by replacing secondary evergreen broadleaf forest with Moso bamboo plantations in subtropical China. Catena (Amst). 2019;180:309–19. 10.1016/j.catena.2019.02.024

[25] Qin H, Niu L, Wu Q, Chen J, Li Y, Liang C, et al. Bamboo forest expansion increases soil organic carbon through its effect on soil arbuscular mycorrhizal fungal community and abundance. Plant Soil. 2017;420:407–21. 10.1007/s11104-017-3415-6

[26] Song Q, Lu H, Liu J, Yang J, Yang G, Yang Q. Accessing the impacts of bamboo expansion on NPP and N cycling in evergreen broadleaved forest in subtropical China. Sci Rep. 2017;7:40383. 28067336 10.1038/srep40383 PMC5220298

[27] Song QN, Ouyang M, Yang QP, Lu H, Yang GY, Chen FS, et al. Degradation of litter quality and decline of soil nitrogen mineralization after moso bamboo (Phyllostachys Pubscens) expansion to neighboring broadleaved forest in subtropical China. Plant Soil. 2016;404:113–24. 10.1007/s11104-016-2835-z

[28] Chen ZH, Li YC, Chang SX, Xu QF, Li YF, Ma ZL, et al. Linking enhanced soil nitrogen mineralization to increased fungal decomposition capacity with Moso bamboo invasion of broadleaf forests. Sci Total Environ. 2021;771:144779. 33736125 10.1016/j.scitotenv.2020.144779

[29] Wu C, Mo Q, Wang H, Zhang Z, Huang G, Ye Q, et al. Moso bamboo (Phyllostachys edulis (Carriere) J. Houzeau) invasion affects soil phosphorus dynamics in adjacent coniferous forests in subtropical China. Ann Sci. 2018;75:24. 10.1007/s13595-018-0703-0

[30] Chen X, Chen HYH, Chang SX. Meta-analysis shows that plant mixtures increase soil phosphorus availability and plant productivity in diverse ecosystems. Nat Ecol Evol. 2022;6:1112–21. 35760890 10.1038/s41559-022-01794-z

[31] Sun H, Hu W, Dai Y, Ai L, Wu M, Hu J et al. Moso bamboo (Phyllostachys edulis (Carrière) J. Houzeau) invasion affects soil microbial communities in adjacent planted forests in the Lijiang River basin, China. Front Microbiol. 2023;14. 10.3389/fmicb.2023.1111498 PMC9990415 36896433

[32] Yang G, Zhou D, Wan R, Wang C, Xie J, Ma C, et al. HPLC and high-throughput sequencing revealed higher tea-leaves quality, soil fertility and microbial community diversity in ancient tea plantations: compared with modern tea plantations. BMC Plant Biol. 2022;22:239. 35550027 10.1186/s12870-022-03633-6 PMC9097118

[33] Martucci do Couto G, Eisenhauer N, Batista de Oliveira E, Cesarz S, Patriota Feliciano AL, Marangon LC. Response of soil microbial biomass and activity in early restored lands in the northeastern Brazilian Atlantic Forest. Restor Ecol. 2016;24:609–16. 10.1111/rec.12356

[34] Beheshti M, Etesami H, Alikhani HA. Interaction study of biochar with phosphate-solubilizing bacterium on phosphorus availability in calcareous soil. Arch Agron Soil Sci. 2017;63:1572–81. 10.1080/03650340.2017.1295138

[35] Lin YT, Whitman WB, Coleman DC, Jien SH, Chiu CY. Cedar and bamboo plantations alter structure and diversity of the soil bacterial community from a hardwood forest in subtropical mountain. Appl Soil Ecol. 2017;112:28–33. 10.1016/j.apsoil.2017.01.001

[36] Lin Y, Te, Tang SL, Pai CW, Whitman WB, Coleman DC, Chiu CY. Changes in the soil bacterial communities in a cedar plantation invaded by moso bamboo. Microb Ecol. 2014;67:421–9. 24072077 10.1007/s00248-013-0291-3

[37] Fu C, Zhu Q, Yang G, Xiao Q, Wei Z, Xiao W. Influences of extreme weather conditions on the carbon cycles of bamboo and tea ecosystems. Forests. 2018;9:629. 10.3390/f9100629

[38] Manawasinghe I. Microfungi associated with Camellia sinensis: a case study of leaf and shoot necrosis on tea in fujian, China. Mycosphere. 2021;12:430–518. 10.5943/mycosphere/12/1/6

[39] Liang A, Wen X, Yu W, Su S, Lin Y, Fan H, et al. Impacts of different reforestation methods on fungal community and nutrient content in an ex-tea plantation. Forests. 2023;14:432. 10.3390/f14020432

[40] Song X, Zhou G, Jiang H, Yu S, Fu J, Li W, et al. Carbon sequestration by Chinese bamboo forests and their ecological benefits: assessment of potential, problems, and future challenges. Environ Reviews. 2011;19 NA:418–28. 10.1139/a11-015

[41] Li Z, Li Z. Mapping the spatial distribution of tea plantations using high-spatiotemporal-resolution imagery in northern zhejiang, China. Forests. 2019;10:856. 10.3390/f10100856

[42] Gao SL, Hu SS, He P, Feng K, Pan RY, Zhang S, et al. Effects of reducing chemical fertilizer on the quality components of Tieguanyin tea leaves. IOP Conf Ser Earth Environ Sci. 2020;559:012020. 10.1088/1755-1315/559/1/012020

[43] Cao Y, Ding S, Qin Y, He X, Ma J. Effects of bamboo-tea mixed model on surface soil organic carbon storage and components. Guihaia. 2022;43:1668–77.

[44] Hou X, Han H, Tigabu M, Cai L, Meng F, Liu A, et al. Changes in soil physico-chemical properties following vegetation restoration mediate bacterial community composition and diversity in Changting, China. Ecol Eng. 2019;138:171–9. 10.1016/j.ecoleng.2019.07.031

[45] Buysse J, Merckx R. An improved colorimetric method to quantify sugar content of plant tissue. J Exp Bot. 1993;44:1627–9. 10.1093/jxb/44.10.1627

[46] Lu RK. Soil agricultural chemical analysis methods. Beijing: China Agricultural Science and Technology; 2000.

[47] Caporaso JG, Kuczynski J, Stombaugh J, Bittinger K, Bushman FD, Costello EK, et al. QIIME allows analysis of high-throughput community sequencing data. Nat Methods. 2010;7:335–6. 20383131 10.1038/nmeth.f.303 PMC3156573

[48] Zeng Q, Liu D, An S. Decoupled diversity patterns in microbial geographic distributions on the arid area (the Loess Plateau). Catena (Amst). 2021;196:104922. 10.1016/j.catena.2020.104922

[49] Nguyen MN, Dultz S, Picardal F, Bui ATK, Pham QV, Dam TTN, et al. Simulation of silicon leaching from flooded rice paddy soils in the Red River Delta, Vietnam. Chemosphere. 2016;145:450–6. 26694795 10.1016/j.chemosphere.2015.11.104

[50] Fraysse F, Cantais F, Pokrovsky OS, Schott J, Meunier JD. Aqueous reactivity of phytoliths and plant litter: Physico-chemical constraints on terrestrial biogeochemical cycle of silicon. J Geochem Explor. 2006;88:202–5. 10.1016/j.gexplo.2005.08.039

[51] Liu X, Fang P, Xiong Y, Peng Q, Yu Z, Luan F, et al. Assessment of the influence of bamboo expansion on Si pools and fluxes in a disturbed subtropical evergreen broadleaved forest. Catena (Amst). 2022;213:106136. 10.1016/j.catena.2022.106136

[52] Li Z, Cornelis J-T, Linden C, Van Vander E, Delvaux B. Neoformed aluminosilicate and phytogenic silica are competitive sinks in the silicon soil–plant cycle. Geoderma. 2020;368:114308. 10.1016/j.geoderma.2020.114308

[53] Li Z, Zhang L, Deng B, Liu Y, Kong F, Huang G, et al. Mapping the spatial distribution of tea plantations using high-spatiotemporal-resolution imagery in northern zhejiang, China. Environ Sci Pollut Res. 2017;24:24989–99. 10.1007/s11356-017-0186-9 28920141

[54] McClaugherty CA, Pastor J, Aber JD, Melillo JM. Forest litter decomposition in relation to soil nitrogen dynamics and litter quality. Ecology. 1985;66:266–75. 10.2307/1941327

[55] Hu YL, Wang SL, Zeng DH. Effects of single Chinese fir and mixed Leaf litters on Soil Chemical, Microbial properties and Soil enzyme activities. Plant Soil. 2006;282:379–86. 10.1007/s11104-006-0004-5

[56] González I, Sixto H, Rodríguez-Soalleiro R, Oliveira N. Nutrient contribution of Litterfall in a short Rotation Plantation of pure or mixed plots of Populus alba L. and Robinia pseudoacacia L. Forests. 2020;11:1133. 10.3390/f11111133

[57] Zou N, Shi W, Hou L, Kronzucker HJ, Huang L, Gu H, et al. Superior growth, N uptake and NH4 + tolerance in the giant bamboo Phyllostachys edulis over the broad-leaved tree Castanopsis fargesii at elevated NH4 + may underlie community succession and favor the expansion of bamboo. Tree Physiol. 2020;40:1606–22. 32816018 10.1093/treephys/tpaa086

[58] Husson O. Redox potential (eh) and pH as drivers of soil/plant/microorganism systems: a transdisciplinary overview pointing to integrative opportunities for agronomy. Plant Soil. 2013;362:389–417. 10.1007/s11104-012-1429-7

[59] Jones RT, Robeson MS, Lauber CL, Hamady M, Knight R, Fierer N. A comprehensive survey of soil acidobacterial diversity using pyrosequencing and clone library analyses. ISME J. 2009;3:442–53. 19129864 10.1038/ismej.2008.127 PMC2997719

[60] Araujo JF, de Castro AP, Costa MMC, Togawa RC, Júnior GJP, Quirino BF, et al. Characterization of Soil Bacterial assemblies in Brazilian Savanna-Like Vegetation reveals Acidobacteria Dominance. Microb Ecol. 2012;64:760–70. 22570118 10.1007/s00248-012-0057-3

[61] Xin W, Zhang J, Yu Y, Tian Y, Li H, Chen X, et al. Root microbiota of tea plants regulate nitrogen homeostasis and theanine synthesis to influence tea quality. Curr Biol. 2024;34:868–e8806. 38366595 10.1016/j.cub.2024.01.044

[62] Xie H, Chen Z, Feng X, Wang M, Luo Y, Wang Y, et al. L-theanine exuded from Camellia sinensis roots regulates element cycling in soil by shaping the rhizosphere microbiome assembly. Sci Total Environ. 2022;837:155801. 35561922 10.1016/j.scitotenv.2022.155801

[63] Tedersoo L, Bahram M, Põlme S, Kõljalg U, Yorou NS, Wijesundera R et al. Global diversity and geography of soil fungi. Science (1979). 2014;346. 10.1126/science.1256688 25430773

[64] Kalwasińska A, Hulisz P, Szabó A, Binod Kumar S, Michalski A, Solarczyk A, et al. Technogenic soil salinisation, vegetation, and management shape microbial abundance, diversity, and activity. Sci Total Environ. 2023;905:167380. 37774878 10.1016/j.scitotenv.2023.167380

[65] Zhang H, Wu X, Li G, Qin P. Interactions between arbuscular mycorrhizal fungi and phosphate-solubilizing fungus (Mortierella sp.) and their effects on Kostelelzkya Virginica growth and enzyme activities of rhizosphere and bulk soils at different salinities. Biol Fertil Soils. 2011;47:543. 10.1007/s00374-011-0563-3

[66] Zhang J, Shen C, Shang TH, Liu JL. Difference responses of soil fungal communities to cattle and chicken manure composting application. J Appl Microbiol. 2022;133:323–39. 35338761 10.1111/jam.15549

[67] Dzurendova S, Zimmermann B, Tafintseva V, Kohler A, Ekeberg D, Shapaval V. The influence of phosphorus source and the nature of nitrogen substrate on the biomass production and lipid accumulation in oleaginous Mucoromycota fungi. Appl Microbiol Biotechnol. 2020;104:8065–76. 32789746 10.1007/s00253-020-10821-7 PMC7447667

[68] Son D, Lee EJ. Associated with three arctic plants in different local environments in Ny–ålesund, Svalbard. J Microbiol Biotechnol. 2022;32:1275–83. 36198667 10.4014/jmb.2208.08009 PMC9668094

[69] Liu J, Sui Y, Yu Z, Shi Y, Chu H, Jin J, et al. Soil carbon content drives the biogeographical distribution of fungal communities in the black soil zone of northeast China. Soil Biol Biochem. 2015;83:29–39. 10.1016/j.soilbio.2015.01.009

[70] Rousk J, Bååth E, Brookes PC, Lauber CL, Lozupone C, Caporaso JG, et al. Soil bacterial and fungal communities across a pH gradient in an arable soil. ISME J. 2010;4:1340–51. 20445636 10.1038/ismej.2010.58

