# Supplementary material for: Soil pH enhancement and alterations in nutrient and Bacterial Community profiles following Pleioblastus amarus expansion in tea plantations
Source: BMC Plant Biol. 2024 Sep 6;24:837. doi: 10.1186/s12870-024-05374-0 (PMC11378374; doi:10.1186/s12870-024-05374-0)
Supplement: Supplementary file 1 — Supplementary Material 1 [file 12870_2024_5374_MOESM1_ESM.docx]

***Supplementary materials***

*Table S1. Basic growth conditions of bamboo and tea trees across various sampling sites after the expansion of Pleioblastus amarus within tea plantations.*

| Sampling sites | *P. amarus* | | | Tea trees | | |  | Elevation/m | Aspect | Slope/° |
| --- | --- | --- | --- | --- | --- | --- | --- | --- | --- | --- |
|  | Density/(clum·hm^-2^) | Mean height /m | Mean DBH /mm | Mean height/m | Crown/m | Number of forks/individual | Band spacing/m |  |  |  |
| BF | 41131.67±5124.53a | 8.83±0.06b | 14.58±0.20b | / | / | / | / | 230-280 | West | 30~35° |
| BA | 48357.50±13277.32a | 9.53±0.75b | 14.53±0.68b | / | / | / | / | 230-280 | West | 30~35° |
| MA | 46273.13±9442.01a | 13.82±0.87a | 21.01±1.35a | 2.10±0.16a | 1.14±0.38a | 8.00±1.55a | 1.40±0.20a | 230-280 | West | 30~35° |
| TB | 41131.67±8073.954a | 14.91±0.47a | 22.37±0.72a | 1.52±0.10b | 0.98±0.08a | 12.33±2.09a | 1.80±0.01a | 230-280 | West | 30~35° |
| TF | / | / | / | 1.13±0.05c | 0.79±0.42a | 1.67±0.06a | 1.67±0.06a | 230-280 | West | 30~35° |

Note: *Different lowercase letters in the same column indicate significant differences at the 0.05 level. The sampling sites are categorized as follows: BF (pure P. amarus forest center area), BA (P. amarus forest interface area), MA (mixed forest center area), TB (mixed forest interface area), and TF (pure tea plantation area).*

*Table S2. Pearson correlation analysis of soil properties and key microorganisms (OUTs) across various sampling sites after to the expansion of P. amarus within tea plantations.*

| Correlation coefficient | pH | OM | TP | TK | HN | AP | AK |
| --- | --- | --- | --- | --- | --- | --- | --- |
| *Mucoromycota* | 0 | -0.92 | 0 | 0 | 0 | 0 | 0 |
| *Patescibacteria* | 0 | 0 | 0 | 0 | -0.91 | 0 | 0 |
| *Chloroflexi* | 0 | -0.89 | 0 | 0 | 0 | 0 | 0 |
| *WPS-2* | -0.91 | -0.88 | 0 | 0 | -0.90 | 0 | -0.90 |
| *Bdellovibrionota* | 0 | 0 | 0 | 0 | -0.93 | 0 | 0 |
| *Firmicutes* | -0.89 | 0 | 0 | 0 | -0.94 | 0 | 0 |
| *Verrucomicrobiota* | 0.89 | 0 | 0 | 0 | 0 | 0.93 | 0 |
| *Methylomirabilota* | 0 | 0 | -0.91 | -0.98 | 0 | 0 | 0 |

*Note: The correlation coefficient was significantly different at the level of 0.05.*


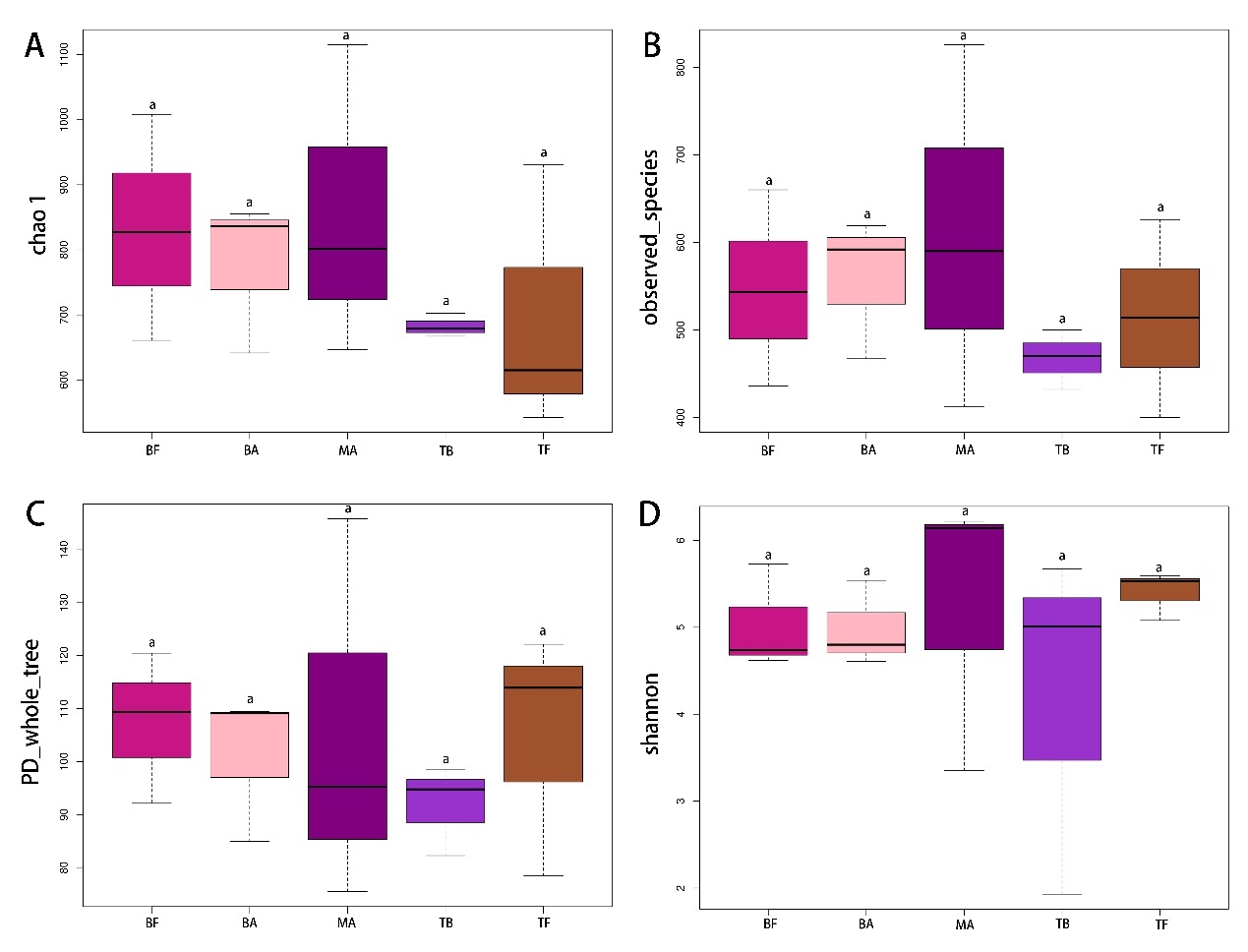


*Figure S1. Soil fungus α-Diversity across various sampling sites after the expansion of P. amarus within tea plantations.*

*Note: Panels A, B, C, and D represent fungus Chao1 index, observed_species, PD_whole_tree, and Shannon index, respectively. Different lowercase letters indicate significant differences in various sampling sites at the 0.05 level. The sampling sites are categorized as follows: BF (pure P. amarus forest center area), BA (P. amarus forest interface area), MA (mixed forest center area), TB (mixed forest interface area), and TF (pure tea plantation area).*


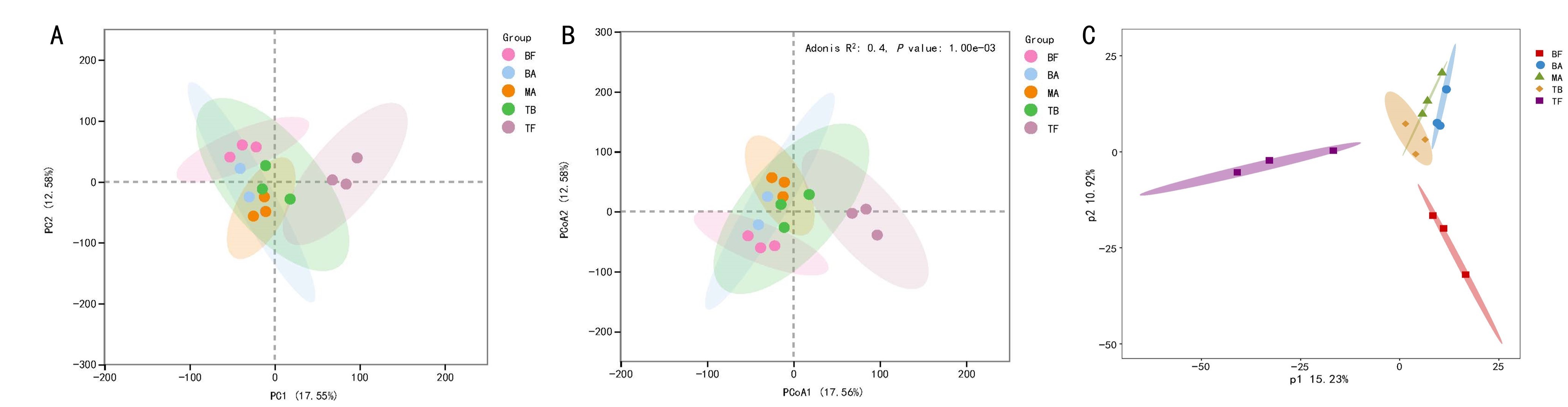


*Figure S2. Soil fungus β-diversity across various sampling sites after the expansion of P. amarus within tea plantations.*

*Note: Panels A, B, and C depict the results of PCA, PCoA, and PLS-DA analyses of fungus OTUs across various sampling sites, respectively. Different colored or shaped dots indicate different sample groups: BF (pure P. amarus forest center area), BA (P. amarus forest interface area), MA (mixed forest center area), TB (mixed forest interface area), and TF (pure tea plantation area).*
